# Acute limb ischemia due to a common iliac artery thrombosis following total pelvic exenteration with pelvic sidewall resection: A case report

**DOI:** 10.1016/j.gore.2025.101750

**Published:** 2025-04-21

**Authors:** Maria Selloua, Mathilde Del, Jean Ségal, Charlotte Chollet, Alejandra Martinez

**Affiliations:** a Department of Surgical Oncology, Institut Claudius Regaud – Institut Universitaire du Cancer de Toulouse (IUCT) – Oncopole, Toulouse, France; b INSERM CRCT Team 1, Tumor Immunology and Immunotherapy, Toulouse, France; c Department of Vascular Surgery, CHU Toulouse, France

## Abstract

Acute limb ischemia following pelvic exenteration with laterally extended endopelvic resection is a rare yet severe complication. Prompt diagnosis is essential for optimal treatment and recovery. This condition should be suspected if the patient presents with rest pain, paresthesia, motor deficit or and sensory loss.

We report a case of a patient, diagnosed with an acute right limb ischemia at day one following a pelvic exenteration with right pelvic sidewall resection for a recurrent cervical cancer after radiotherapy. Through the collaboration of a multidisciplinary team, the patient was successfully treated with surgical thrombectomy.

## Highlights

• Acute limb ischemia caused by common iliac artery thrombosis following total pelvic exenteration and pelvic sidewall resection is a rare but serious complication.
• Prompt diagnostic work-up and intervention is a key factor for limb salvage.
• These complex cases must be managed by a multidisciplinary team in specialized centers.
• Vigilant post-operative monitoring for vascular complications is crucial in patients undergoing complex pelvic surgeries.

## 1 Introduction

According to the ESGO guidelines for cervical cancer, extended pelvic surgery may be considered for the treatment of a locoregional recurrence in patients who have already received radiotherapy (Cibula, 2023). Surgery must aim for a complete tumor resection (R0) with the help of special techniques as laterally extended endopelvic resection (LEER). These complex procedures are performed in specialised centers. During the procedure, the internal iliac vessels are commonly ligated and resected *en bloc* with the tumor, without reconstruction (Daix, 2022). The external and common iliac vessels can be resected in case of involvement, and this procedure must be anticipated and planned with a vascular surgeon, due to the need for reconstruction of the artery (Rajendran et al., 2023). These procedures are associated with a high risk of complications. In a series of 646 patients treated by LEER for a locally advanced and recurrent pelvic malignancy, 16.4 % of patients had major complications, the most common were intra-abdominal collection (43.7 %) and wound infection (14.1 %) (Peacock, 2020). In this study, no case of acute limb ischemia (ALI) was described. To our knowledge, there are no reported cases of ALI following total pelvic exenteration. We report the first case of right limb ischemia due to common iliac artery thrombosis after total pelvic exenteration with right LEER for recurrent cervical cancer.

## 2 Case report

A 47-year-old multiparous woman, with a BMI of 19.6 kg/m^2^ and a history of smoking (ceased two years prior), presented with no medical history other than laparoscopic appendectomy and cholecystectomy. She was diagnosed with stage IV squamous cervical cancer in April 2022, with histologically confirmed mediastinal lymph node involvement. Treatment included six cycles of CARBOPLATIN, PACLITAXEL, and BEVACIZUMAB, followed by BEVACIZUMAB maintenance until June 2022. Persistent local disease in October 2022 led to chemoradiotherapy (CRT) with 45 Gy in 25 sessions and four cycles of CISPLATIN, as well as high-dose rate image-guided brachytherapy (IGBT), with good metabolic response.

**Fig. 1:**
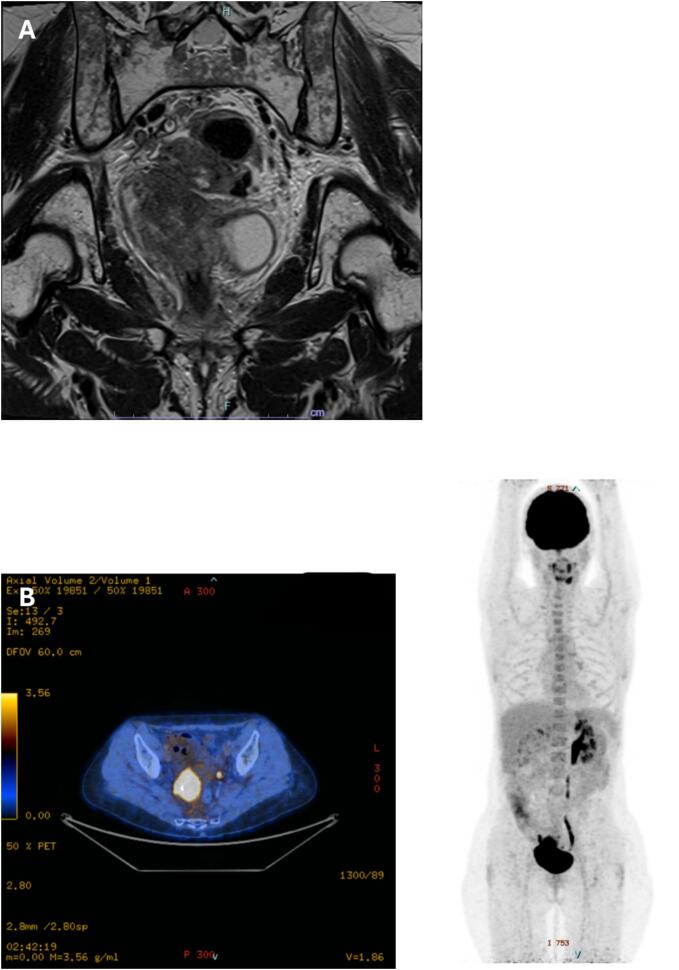
Pre-operative imaging assessment with pelvic MRI (A) and PET-CT (B). A. MRI in axial plane showing the tumor with the lateral involvement. B. PET-CT showing the hypermetabolism of the tumor.

12 months after the completion of CRT and IGBT, an isolated local recurrence was identified, presenting as a 4 cm tumor infiltrating the right parametrium, right ureter, ureterovesical junction, obturator muscle, and rectosigmoid. Imaging indicated 9 cm sidewall involvement on the coronal plane. Close contact with the internal iliac vessels was described, but no thrombosis was present prior to surgery. **(**Fig. 1**)** despite platinum-based chemotherapy and immunotherapy (PEMBROLIZUMAB), the disease persisted, and the tumor board recommended pelvic exenteration with right pelvic sidewall resection

**Fig. 2:**
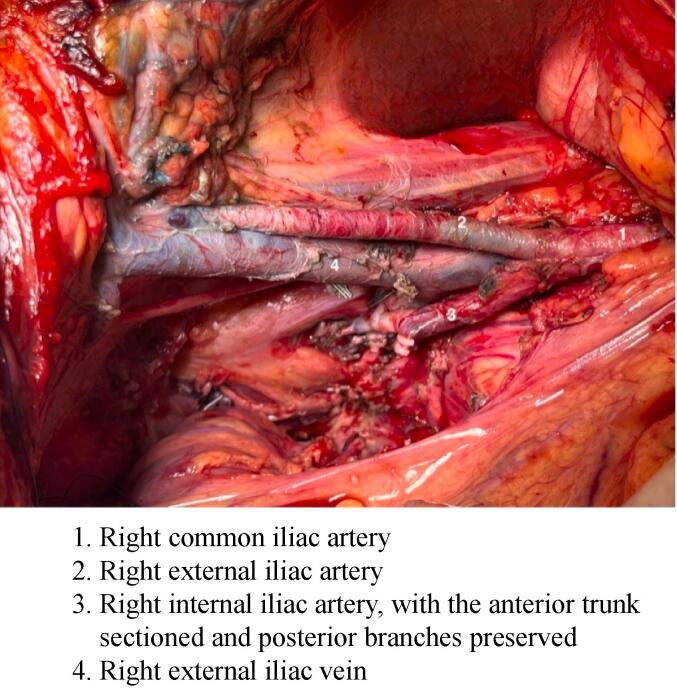
Immediate post operative view of the right pelvic side after the *en bloc* resection of the tumor.

Following thorough counseling about risks and consequences, the patient consented to the surgery, which included total supralevatory exenteration with right pelvic sidewall resection via laparotomy. The procedure entailed sectioning the hypogastric vein and artery while preserving the superior gluteal branch. (Fig. 2) No clamping of the common iliac artery was required. Reconstruction involved a Bricker ileal conduit, pelvic epiplooplasty, left colostomy, and vaginal reconstruction using a deep inferior epigastric perforator (DIEP) flap. A drain was positioned in the pelvis, along with two mono-J ureteral stents.

The patient was positioned in the dorsal decubitus position, with legs slightly apart. She was equipped with a left radial arterial catheter, a central venous port in the left internal jugular vein, and a thoracic epidural catheter. The procedure lasted 520 min, and the blood loss was estimated at 1680 ml. During the procedure, she received one blood unit, 6000 ml in total of ISOFUNDIN, and low-dose NORADRENALIN (mean of 16 µg/ml, administered continuously via an electric syringe pump and discontinued at the end of the procedure). The *peri*-operative biological assessment is shown in Table 1**.**

1. Right common iliac artery
2. Right external iliac artery
3. Right internal iliac artery, with the anterior trunk sectioned and posterior branches preserved
4. Right external iliac vein

**Table 1:** Laboratory workup results and normal ranges.

**Laboratory test**	**Day before surgery**	**End of surgery**	**Day after surgery**	**Normal range and unit**
**Hemoglobin**	10.3	9.1	9.0	12–16 g/dl
**Platelets**	189 000	136 000	142 000	150 – 450 10^3^ G/L
**TP**	96	64	57	70–100 %
**TCA**	0.80	1.16	Non-interpretable*	0.8–1.2 (seconds)
**Fibrinogen**	3.5	2.9	4.0	2.0–4.0 g/L

*The patient was receiving unfractionated heparin.

**Fig. 3:**
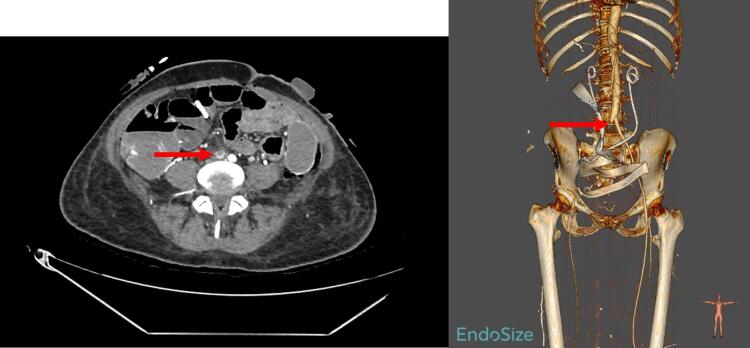
Contrast enhanced CT imaging at diagnosis, showing the right common iliac thrombosis and 3D reconstruction (red arrow).

Postoperatively, the patient used thigh-length intermittent pneumatic compression devices and was admitted to the ICU for close monitoring. Medical prophylaxis with low-molecular-weight heparin (LMWH) was started 6 h post-surgery. Within 24 h, she developed acute right lower limb pain, with signs of peripheral hypoperfusion and sensorimotor deficit on clinical examination: Rutherford Stage III (Björck, 2020). CT imaging revealed a thromboembolism of the right common iliac artery. (Fig. 3) Emergency Fogarty embolectomy and open fasciotomy for compartment syndrome were performed following arteriography. The procedure was followed by 48 h of intravenous antithrombotic therapy using unfractionated heparin (UFH) administered continuously, and monitored by anti-Xa activity, with a target range between 0.3 and 0.6 UI/ml. It was then transitioned to LMWH at prophylactic dosing. Drain removal occurred on day 3, and aponeurotomy closure was performed on day 8.

The patient also developed septic shock due to a pyelonephritis 10 days after revascularization, and was successfully treated by antibiotics alone. She was discharged on day 44. Subsequent CT imaging showed complete reperfusion of the artery. Daily physiotherapy facilitated recovery; however, at the one-month follow-up, the patient still exhibited a 2/5 motor deficit in the right lower limb but was ambulatory and capable of climbing stairs.

Pathological analysis revealed a p16-positive squamous cell carcinoma measuring 60 × 45 mm, infiltrating the vagina, bladder wall, rectal wall, and right ureter. The tumor was in contact with both vaginal and bowel surgical margins. The tumor board recommended active surveillance. Six months later, the patient presented with peritoneal carcinomatosis and is currently receiving weekly treatment with PACLITAXEL.

## 3 Discussion

ALI is a vascular emergency defined by a sudden loss of arterial perfusion, that threatens limb viability, requiring urgent evaluation and treatment (within 6 h) to prevent irreversible damage or amputation (Acar et al., 2013). Its etiology includes embolism, thrombosis, or iatrogenic causes, such as surgical trauma or hypercoagulable states (Costa, 2024). Clinically, ALI manifests with rest pain, paresthesia, motor deficit, and sensory loss, with mortality and amputation rates reaching 15 % and 25 %, respectively, at 30 days (Rajan, 2009). In the postoperative setting, ALI presents a diagnostic challenge, requiring high vigilance and rapid management.

The patient presented with a postoperative thromboembolism of the right common iliac artery following total pelvic exenteration with LEER. The complexity of this surgical intervention, compounded by prior CRT and chronic tumor compression, significantly increased the risk of vascular complications. Radiotherapy induces arterial fibrosis and endothelial damage, contributing to thrombotic risk (Korpela and Liu, 2014). Additionally, extensive surgical manipulation of vascular structures and prolonged operative times further predispose patients to ALI.

Postoperative ALI often results from embolization, vascular injury, or iatrogenic factors, as seen in this case. Early diagnosis is critical and relies on clinical examination, with an ankle-brachial index (ABI) < 0.90, and imaging modalities such as CT angiography (CTA) or Doppler ultrasound to confirm the extent of arterial compromise (Aboyans, 2012). This patient’s prompt identification of ALI and timely intervention with Fogarty embolectomy and fasciotomy underscore the importance of early action in reducing morbidity and preserving limb function. Management strategies depend on the duration and severity of ischemia. Catheter-directed thrombolysis is effective for recent thrombotic events but is contraindicated in patients with recent major surgery due to bleeding risks or in those with neurologic symptoms, due to its delayed action. In this case, surgical thromboembolectomy was performed, followed by anticoagulation therapy to prevent recurrent thrombosis. Prophylactic measures, such as atraumatic vascular handling and avoiding prolonged vessel compression during surgery, help reduce the risk of such complications. However, vessel dissection in cases of previously irradiated large latero-pelvic tumor relapses presents a surgical challenge. Close postoperative monitoring, particularly in high-risk patients, is essential for the early detection and intervention of ALI to restore perfusion and prevent amputation. Special attention must be given to those patients requiring a complementary epidural anesthesia which can dull the main symptom, pain.

In this case, ICU observation facilitated rapid diagnosis and intervention, likely prevented limb loss. The use of UFH or LMWH for venous thromboembolism prophylaxis during the perioperative period is effective in reducing deep venous thromboembolism complications in gynecologic oncologic surgery, but does not prevent arterial thrombosis (Key, 2020). Future preventive strategies may include prophylactic antiplatelet therapy in selected high-risk patients, guided by multidisciplinary evaluation. Secondly, early and regular lower limb clinical examination with pulse check after surgeries involving LEER could help improve the time of management, which is a key prognostic factor.

The rarity of such complications highlights the need for standardized protocols to prevent and manage postoperative ALI. Surgical teams must be trained to promptly identify and manage vascular emergencies, with an emphasis on comprehensive care protocols that include early detection, appropriate imaging, and timely intervention.

## 4 Conclusion

This case illustrates ALI resulting from common iliac artery thrombosis, as a rare but life-threatening complication following total pelvic exenteration with LEER. Early detection through vigilant postoperative monitoring and swift intervention with embolectomy and anticoagulation enabled limb salvage and recovery. The patient’s favorable outcome underscores the importance of close postoperative monitoring, rapid intervention, and prevention strategies, such as meticulous surgical techniques and prophylactic antiplatelet therapy for well-selected high-risk patients.

Written informed consent was obtained from the patient for publication of this case report and accompanying images.

## CRediT authorship contribution statement

**Maria Selloua:** Writing – original draft, Methodology, Data curation, Conceptualization. **Mathilde Del:** Writing – original draft, Methodology, Data curation, Conceptualization. **Jean Ségal:** Methodology, Data curation, Conceptualization. **Charlotte Chollet:** Methodology, Data curation. **Alejandra Martinez:** Supervision, Methodology, Data curation, Conceptualization.

## Declaration of competing interest

The authors declare that they have no known competing financial interests or personal relationships that could have appeared to influence the work reported in this paper.
